# Frontal EEG Asymmetry and Middle Line Power Difference in Discrete Emotions

**DOI:** 10.3389/fnbeh.2018.00225

**Published:** 2018-11-01

**Authors:** Guozhen Zhao, Yulin Zhang, Yan Ge

**Affiliations:** ^1^CAS Key Laboratory of Behavioral Science, Institute of Psychology Beijing, China; ^2^Department of Psychology, University of Chinese Academy of Sciences Beijing, China

**Keywords:** frontal EEG asymmetry, midline power, discrete emotion, valence, arousal, film clip

## Abstract

A traditional model of emotion cannot explain the differences in brain activities between two discrete emotions that are similar in the valence-arousal coordinate space. The current study elicited two positive emotions (amusement and tenderness) and two negative emotions (anger and fear) that are similar in both valence and arousal dimensions to examine the differences in brain activities in these emotional states. Frontal electroencephalographic (EEG) asymmetry and midline power in three bands (theta, alpha and beta) were measured when participants watched affective film excerpts. Significant differences were detected between tenderness and amusement on FP1/FP2 theta asymmetry, F3/F4 theta and alpha asymmetry. Significant differences between anger and fear on FP1/FP2 theta asymmetry and F3/F4 alpha asymmetry were also observed. For midline power, midline theta power could distinguish two negative emotions, while midline alpha and beta power could effectively differentiate two positive emotions. Liking and dominance were also related to EEG features. Stepwise multiple linear regression results revealed that frontal alpha and theta asymmetry could predict the subjective feelings of two positive and two negative emotions in different patterns. The binary classification accuracy, which used EEG frontal asymmetry and midline power as features and support vector machine (SVM) as classifiers, was as high as 64.52% for tenderness and amusement and 78.79% for anger and fear. The classification accuracy was improved after adding these features to other features extracted across the scalp. These findings indicate that frontal EEG asymmetry and midline power might have the potential to recognize discrete emotions that are similar in the valence-arousal coordinate space.

## Introduction

Emotion plays an important role in communication and daily interpersonal events (Darwin and Prodger, 1998). Emotion is an affective state of human beings as well as animals that arises in response to the perception of an object or a situation (Verma and Tiwary, 2014). Emotions can be regarded as episodes of interrelated, synchronized changes in the following components: cognitive processing, subjective feeling, action tendencies, physiological changes and motor expression (Kipp and Martin, 2009). Additionally, determining the differences among emotions in relation to physiological changes and subjective feelings is rather important. Although there is no commonly agreed-upon definition of emotion and giving it a precise and complete definition is difficult (Mulligan and Scherer, 2012), researchers have attempted to describe emotions generally from two perspectives, the dimensional model and discrete model of emotion.

The dimensional model confirms that emotions can be represented by combinations of a few basic and fundamental dimensions. Researchers overwhelmingly agree that two basic dimensions, valence and arousal, are required to describe emotions, which is also called a “circumplex model” (Lang et al., 1993). Specifically, the valence level ranges from unpleasant (negative) to pleasant (positive) and the arousal level ranges from not aroused (low arousal) to excited (high arousal).

**Figure 1:**
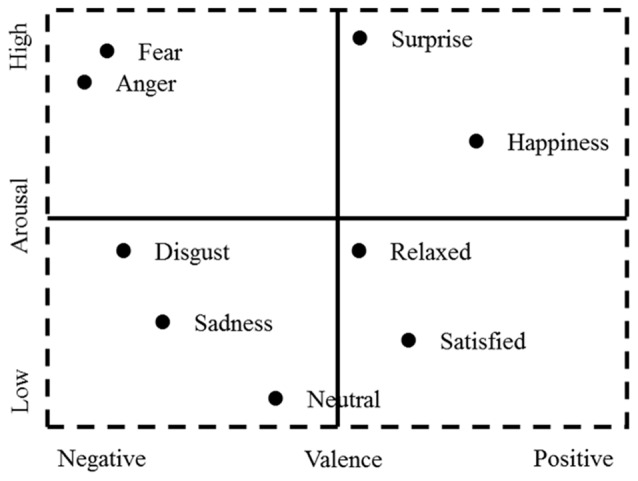
2D emotion model by valence and arousal.

The other model is the discrete model of emotion, which states that the emotion space comprises limited discrete basic emotions and complex emotions, which are a combination of basic emotions (Barrett et al., 2007). The number of basic emotions remains slightly controversial, but there is a consensus regarding the following basic emotions: anger, fear, sadness, happiness, disgust and surprise (Ortony and Turner, 1990; Panksepp, 2010; Barrett, 2011; Ekman and Cordaro, 2011). Recently, researchers have tried to represent different discrete basic emotions using a dimensional pattern to integrate these two models (Mauss and Robinson, 2009; Hamann, 2012; Hu et al., 2017; Liu et al., 2017). Accordingly, both anger and fear can be described as “negative valence” and “high arousal, ” whereas satisfied can be characterized as “positive valence” and “low arousal” (see Figure 1; Javela et al., 2008; Barrett, 2012; Lindquist et al., 2012).

Nevertheless, differentiating between two discrete emotions that are similar in the valence and the arousal dimension is difficult. When two or more discrete emotions are close in the valence-arousal coordinate space, the recognition ability will be degraded dramatically (Fontaine et al., 2007). One possible reason is that emotions of similar valence and arousal dimensions (such as anger and fear) might display their own unique representation and further induce completely different behaviors (Yin et al., 2014). Notably, emotion is a psycho-physiological process, and individuals display differential heart rates, blood pressure, peripheral vascular resistance responses and brain activities when they experience different emotional states (Bradley et al., 2001; Kop et al., 2011). Electroencephalographic (EEG) signals are not easily disguised, occur in real time and are sensitive to emotional changes, which means that they can measure the brain’s immediate responses to affective stimuli in real time (Bekkedal et al., 2011). Discrete emotional categories correspond to different EEG activities. For example, the emotions of happiness, joy, anger, disgust, fear/anxiety and sadness were reportedly characterized by their own individual patterns in the distribution of the amplitude-frequency characteristics of the EEG (Aftanas et al., 2006). Therefore, one state-of-the-art real-time emotion recognition system from EEG signals has been proposed to distinguish two similar emotions in the valence-arousal coordinate space (Liu et al., 2017). Additionally, two major EEG features, frontal asymmetry and midline power, may serve important roles.

Frontal asymmetry is a typical indicator of asymmetric brain activity in the frontal cortex, which refers to asymmetrical activity between the left hemisphere and right hemisphere (Briesemeister et al., 2013). In previous studies, emotions in similar valence-arousal coordinate spaces, such as fear and anger (Barrett, 2012), were associated with different frontal EEG asymmetries (Coan et al., 2001). Evidence from neuroimaging has found that fear and anger are associated with different anatomic pathways (Labar and LeDoux, 1996). According to other studies, changes in the activation of the frontal lobes in response to pictures of anger were different from those in response to pictures of fear (Balconi and Pozzoli, 2009). In addition, higher levels of left frontal activity and lower right frontal activity were observed when individuals were insulted and felt angry, which would further lead to aggressive behaviors (Harmon-Jones and Sigelman, 2001). For fear, greater right frontal activity could predict the experience of fear during the presentation of a toy spider to an infant (Diaz and Bell, 2012).

Two frequency bands, theta and alpha, are closely related to frontal asymmetry. Theta band (4–8 Hz) waves are first observed during sleep and are relevant to the arousal level, and theta waves exist during tasks that require the correlation of increased mental effort and sustained concentration (Sammler et al., 2007). Alpha band (8–13 Hz) waves exist when a person is in relaxation mode, and they may reflect the progress of perceptual processing, memory tasks, and the processing of emotions (Sanei and Chambers, 2007). Alpha power is considered to be inversely related to regional brain activity, and decreased power values of the alpha band indicate an increase in cortical activation (Allen et al., 2004). In the early stage, frontal asymmetry was regarded as a reflection of valence. A relative increase in left hemisphere activity was observed with positive emotional stimuli, whereas greater right hemisphere activity was associated with negative emotions (Davidson and Henriques, 2000; Balconi and Mazza, 2010; Poole and Gable, 2014). Previous studies also revealed that positive stimuli such as pleasant odors, happy musical excerpts and pleasant advertisements could significantly induce lower frontal alpha power and higher theta power in the left hemisphere (Kline et al., 2000; Schmidt and Trainor, 2001; Vecchiato et al., 2011). However, there was more evidence to support the view that the frontal alpha asymmetry was more closely related to the motivation dimension. The approach-withdrawal model confirmed that greater right than left frontal alpha power (left-hemispheric activation) was linked to approach-related motivation (Poole and Gable, 2014). Frontal asymmetry was also correlated with other dimensions in addition to valence and arousal, such as self-reported dominance (Reuderink et al., 2013). As a result, although anger and fear are similar in the valence-arousal coordinate space, it is still possible to differentiate them by analyzing the EEG activity at the frontal cortical level, which might reflect other dimensions.

Another EEG feature, midline power, especially frontal midline theta, was also associated with emotional processing (McFarland et al., 2016). Frontal midline theta was suggested as a better candidate than frontal alpha activity for use in a BCI-based paradigm designed to modify emotional reactions (McFarland et al., 2016). Frontal midline theta was considered to be associated with positive emotional experience and the relaxation state from anxiety (Suetsugi et al., 2000). For example, the intensity of blissful experience was reportedly positively related to theta power in the anterior frontal and frontal midline sites (Aftanas and Golocheikine, 2001).

Other frequency bands in addition to theta were also related to the dimension of tension and relaxation. For example, beta power, which is responsible for peoples’ concentration level, was positively related to tension (Mao, 2013). Additionally, an increase in theta power, particularly in the frontal medial and central medial area, as well as a greater reduction in alpha power, was observed during relaxation (Sandler et al., 2016). Thus, it is possible to differentiate two emotions that are similar in valence and arousal using the primal EEG features discussed above.

Notably, previous research offers few straightforward hypotheses for the EEG difference between positive emotions. Both tenderness and amusement induced by film clips showed a moderate level of arousal according to subjective rating scores (Schaefer et al., 2010). Tenderness as an affiliative positive emotion has received less attention than other typical positive emotions such as amusement and joy (Takahashi et al., 2008). Tenderness is defined as a momentary experience corresponding to love as caregiving, and it is always accompanied by an expansive “warm-and-fuzzy” feeling (Dijker, 2010; Kalawski, 2010). Compared with other positive emotions, tenderness showed different peripheral physiological responses. For example, tenderness decreased heart rate, but joy increased heart rate (Santibanez and Bloch, 1986). However, the differences between tenderness and other positive emotions in brain activities remain unknown. Tenderness was related to subjective feelings of acceptance, gratifying pleasure and warmth-liking, and it was induced by proximal affiliative stimuli (Depue and Morrone-Strupinsky, 2005). Compared with amusement, which is evoked by humorous material, tenderness is more related to a fundamental motivational ‘care-liking’ system, which motivates affiliation and caring, subserving the goal of attachment (Schweiger et al., 2014). Thus, tenderness might be more correlated with consummatory positive affect, such as liking and approach tendency, which can be reflected by frontal asymmetry. Additionally, greater left hemisphere activation might be observed in tenderness than in amusement, which must be explored in the current study.

Taken together, the traditional models of emotion cannot explain the difference between two similar emotions in the valence-arousal coordinate space. As different discrete emotional states may correlate with different EEG activities, the aim of the present study was to examine the differences in the EEG activities between two positive emotions and between two negative emotions similar in both valence and arousal dimensions. Amusement as a typical positive emotion and tenderness as an overlooked positive emotion were selected for the current study. Alternatively, many existing studies have focused on the difference between anger and fear in both valence and arousal dimensions (Wacker et al., 2003; Harmon-Jones, 2007; Liu et al., 2014). The current study aimed to elicit these two negative emotions that are similar in the valence-arousal coordinate space and then differentiate fear from anger in addition to the valence and arousal dimensions (Ellsworth and Scherer, 2003). Moreover, the EEG-based recognition of different affective states has raised great concern, and previous results showed that positive and negative affective states could be distinguished successfully using EEG powers from all channels (Nie et al., 2011; Stikic et al., 2014). However, no study has explored the recognition of two emotions within valence and arousal according to the EEG correlates with different emotional states. Thus, another aim of the current study was to classify two emotions that are similar in both valence and arousal using both midline and frontal EEG power features and compare the effectiveness of using these new features with other previously used EEG features.

## Materials and Methods

### Participants

Thirty-three (16 males and 17 females) healthy undergraduate or graduate students without neurological illness or psychiatric disorders participated in the current study. Participants ranged in age from 18 years to 26 years (mean = 23.85, *SD* = 2.57). All participants were right-handed and had normal or corrected-to-normal visual acuity. This study was carried out in accordance with the recommendations of Guildline of Human Experimentation, Institutional Review Board of the Institute of Psychology, Chinese Academy of Sciences. The protocol was approved by the Institutional Review Board of the Institute of Psychology, Chinese Academy of Sciences. All subjects gave written informed consent in accordance with the Declaration of Helsinki.

### Stimulus Material

Stimulus materials were four movie clips taken from the standard Chinese emotional film clips database (Liu et al., 2017) and applied to the following target emotions: amusement (“Just Another Pandora’s Box” presents a humorous battle scene, 67 s in length), tenderness (“A Simple Life” recalls the master’s happy childhood, 99 s in length), anger (“City of Life and Death” describes the scene of the Nanjing massacre, 73 s in length), and fear (“Inner Senses” shows a ghastly scene where the hero sees a ghost, 92 s in length). All the four movie clips used in the current study have been proven effective in eliciting their respective target emotions, according to our previous study (Liu et al., 2017). Specifically, each movie clip showed a combination of the highest-hitting rate and target-rating scores, demonstrating high ecological validity.

### Self-Assessment Scales

Self-assessment scales were modified from a 9-point Likert self-assessment manikin (SAM; Bradley and Lang, 1994). SAM measures the extent to which participants experience the dimensions of arousal, valence, liking, familiarity and dominance while viewing the movie clips (1 = “not at all”, 5 = “moderately”, 9 = “extremely”). In addition, a 4-word differential emotions scale (DES, amusement, tenderness, anger and fear, on a 9-point Likert scale, 1 = “not at all”, 5 = “moderately”, 9 = “extremely”) was appended to the self-assessment scales. Participants were told to report their true feelings while viewing the movie clips and then finish the self-assessment scales.

### Experimental Procedure

**Figure 2:**
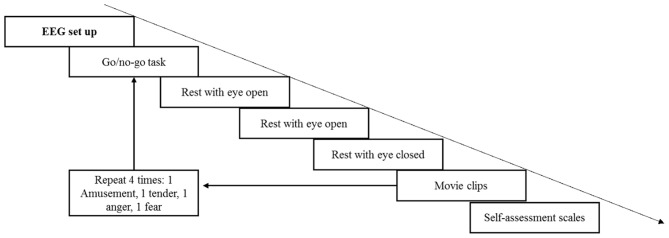
The timing diagram of the experiment.

First, the participants provided informed consent and were given introductions to the current study to ensure that they knew the entire procedure and what they should do. Next, subjects were seated comfortably in a dimly lit, electrically shielded room with the monitor screen positioned approximately 60 cm in front of their eyes. The EEG setup was accomplished with the help of two assistants; during the setup, the participants were instructed to relax and remain calm in preparation for the upcoming experiment. The volumes of two speakers were set at a relatively loud level. Similar to Koelstra et al. (2012), each participant was asked before the experiment whether the volume was comfortable, and all the participants reported that it was appropriate. In the formal experiment, the volume remained fixed to equate auditory stimuli among the four movie clips. The entire experiment comprised four trials (see Figure 2). During each trial, the participants were required to finish a 60-s go/no go task to keep them in a neutral emotional state. The participants were asked to press a button when number 1 was presented and not to press the button when number 9 was presented on the screen. The presentation probability of number 1 was 20%, and the inter-stimulus intervals (ISI) was 1,200–1,800 ms. Then, a rest period with eyes open for 40 s and eyes closed for another 40 s was arranged to record the baseline EEG data. Next, four movie clips were shown to the participants in random order. The participants were asked to watch the movie clips and fill out the rating scales carefully. Each film clip was adjusted to the same resolution (720 × 576) without subtitles. During the entire experiment, EEG signals were recorded, and the participants were asked to keep their chin on the chin strap except during the rest period.

### EEG Data Acquisition and Analysis

EEG signals were recorded with a NuAmps 40-channel monopolar DC amplifier system (NeuroScan Inc., Charlotte, NC, USA) with sampling at 1,000 Hz and a 22-bit resolution. An Electro Cap with 32 active Ag/AgCl electrodes was used to record EEG from active scalp sites referred to as the 10/20 system of electrode placement (FPZ, FP1, FP2, F7, F3, FZ, F4, F8, FT7, FC3, FCZ, FC4, FT8, T3, C3, CZ, C4, T4, TP7, CP3, CPZ, CP4, TP8, T5, P3, PZ, P4, T6, O1, OZ and O2). A ground electrode was mounted at the center of the forehead (midway between FPZ and FZ). Four EOG electrodes were placed on the outer side of the eyes (two horizontal electrodes at the outer canthus of both eyes and two vertical electrodes above and below the left eye) to reject ocular artifacts. The impedance of the recording electrodes was monitored for each subject before data collection, and it was maintained below 5 kΩ.

### Data Analysis

Neuroscan software 4.5 was used in the current study to process EEG data. The data were recorded using a sampling rate of 500 Hz with a frequency band of 1–35 Hz. Then, the data were segmented into epochs with artifact voltages exceeding the threshold, which varied among the participant population. Ocular artifacts were removed from the raw EEG data using a regression procedure implemented in Neuroscan software. Clean EEG data were re-referenced to the algebraic mean of the left mastoids and right mastoids to obtain a symmetric reference. Artifacts such as aberrant signals were visually checked and removed by hand. Artifact-free EEG epochs were selected and transformed into a frequency domain by short-term Fourier transformation through a 2-s Hanning window with an overlap of 50% to reduce spectral leakage and minimize data loss. Mean band power values within the theta band (4–8 Hz), alpha band (8–13 Hz) and beta bands (13–30 Hz) were calculated by averaging the power values across frequency bins. To reduce the interindividual variance of absolute power values and the confounding effect of the previous film clip on the current one, we normalized the power values within three bands using the baseline spectra according to a formula used in previous research (Sammler et al., 2007). Specifically, for each participant, each emotion category (C), electrode (e), and frequency band (f), the individual band power PC (e, f) was divided by the average band power PB (e, f) across *N* = 30 scalp electrodes measured in the corresponding baseline (B) before each movie clip with eyes open and in the same frequency band (f):

$${\hat{P}}_{C}(e,f)=\frac{P_{C}(e,f)}{\frac{1}{N}*\sum_{e=1}^{N}P_{B}(e,f)}$$

According to a previous study, alpha bands at FP1/FP2 and F3/F4 are effective in discerning emotional states with 90% confidence (Yoon and Chung, 2011). For frontal asymmetry, consistent effects were observed at the F3/F4 site (Harmon-Jones and Sigelman, 2001; Wacker et al., 2013). In addition, according to previous results, the maximal frontal midline theta was found at Fz and F3/F4 (Mitchell et al., 2008), and it was localized to Fcz (Sammler et al., 2007). In the current study, F3/F4 was included in the frontal localization (FP1/FP2 and F3/F4), and midline sites were extended to the parietal lobe (F_Z_, FC_Z_, C_Z_, CP_Z_ and P_Z_) to explore the trend.

First, descriptive statistics and gender differences were analyzed. Then, several two-way repeated measures MANOVAs were conducted for the average power spectra of theta and alpha bands, with emotion category (amusement and tenderness) and hemisphere (left and right) as two within-subject variables at frontal sites (FP1/FP2, F3/F4). When an interaction or a main effect was significant, *post hoc* comparisons were performed with a Bonferroni correction. Additionally, several paired-samples *t* tests were conducted for the average power of theta, alpha and beta bands between two positive emotions and two negative emotions at midline sites (F_Z_, FC_Z_, C_Z_, CP_Z_ and P_Z_).

Then, to test the relationship between EEG features and the subjective rating of target emotions, we conducted regression and partial correlation analysis. Stepwise multiple regression analyses were performed to predict the subjective ratings of four target emotions with EEG features as independent variables. Partial correlations were also conducted to show the relations between EEG features and other subjective ratings. The level of the type 1 error was set to *p* < 0.05.

Finally, machine-learning algorithms were applied to investigate the contribution of EEG asymmetry and middle line power features in the classification of two positive and two negative emotions. First, different power features extracted from EEG signals were collected as the pool of candidate features. Then, sparse linear discriminant analysis (SLDA) was applied to rank the candidate features (Sjöstrand et al., 2018). The basic idea of linear discriminant analysis is to project the high-dimensional pattern samples into the optimal discriminant vector space to extract the classification information and compress the dimension of feature space. SLDA seeks discriminant vectors (w_1_, w_2_, …, w_k-1_), which successively maximize the between-class variance relative to its within-class variance. SLDA adds a sparseness constraint to all discriminant vectors, which can make many elements in the discriminant vectors *w*_i_ exactly zero. The remaining features corresponding to nonzero elements in *w*_i_ can be ranked by the absolute value of these nonzero elements and were selected for recognizing tenderness and amusement. The selected features were entered as inputs to a support vector machine (SVM) classifier, which seeks to separate different classes of examples apart with maximum margin for the hyperplane (Liu et al., 2017; Zhao et al., 2017). The leave-one-subject-out subject-independent cross validation was utilized, and the average classification accuracy was computed. Specifically, recorded trials from all but one subject was used to train the algorithm, which was then tested on the remaining subject. The procedure was repeated as many times as there were subjects, and the results were averaged across all subjects (Novak et al., 2014).

## Results

### Subjective Ratings

The validity of each film’s target emotion was assessed by the hit rate, which is defined as the proportion of reviewers who rate the target emotion score at least one point higher than the non-target emotion (Gross and Levenson, 1995). Each film’s anticipated target emotion of all 33 participants received a higher rating (at least 1 point) than the other three emotional categories. In addition, all four movies successfully elicited target emotions with a hit rate of 100%. The average target-rating scores for four movies are shown in Table 1. In addition, the subjective assessment of arousal, valence, liking, familiarity and dominance is shown in Table 1.

**Table 1:** Subjective assessment of arousal, valence, liking, familiarity and dominance [M (SD)].

	Arousal	Valence	Liking	Familiarity	Dominance	Target score
Amusement	5.94 (2.12)	5.91 (2.37)	5.14 (2.47)	2.24 (2.27)	5.78 (2.23)	6.84 (2.09)
Tenderness	5.39 (1.52)	5.30 (1.74)	5.38 (1.99)	2.73 (2.42)	6.51 (1.97)	7.22 (1.64)
Anger	7.46 (1.50)	1.09 (0.29)	1.49 (1.12)	2.95 (2.77)	4.03 (2.40)	7.70 (1.53)
Fear	7.30 (1.81)	1.24 (0.44)	2.11 (1.70)	1.24 (0.76)	3.68 (2.42)	6.65 (2.36)

Paired sample *t* tests were conducted to test whether the two positive or negative emotions were similar on the arousal and valence scores. For the two positive emotions, the rating of amusement on arousal was nonsignificant between amusement and tenderness (*t* = 1.67, *p* = 0.104). Additionally, the difference in valence was nonsignificant (*t* = 1.58, *p* = 0.125). In regard to the two negative emotions, the rating on arousal was nonsignificant between anger and fear (*t* = 0.21, *p* = 0.839). Additionally, the difference in valence was significant (*t* = −0.21, *p* = 0.044). Similar to the definition of the hit rate, the frequency of participants who rated the valence of the fear film one point higher than that of the anger film was calculated. Only three participants rated the valence of the fear film one point higher than that of the anger film. There was a significant difference between these two frequencies ($\chi_{\left(1\right)}^{2}$) = 22.09, *p* < 0.05). Therefore, the frequency of participants who rated anger and fear as similar on valence was significantly greater than that of the remaining participants. Therefore, two positive emotions and two negative emotions were similar on both the arousal and valence dimensions.

Paired sample *t* tests were also performed on the liking, familiarity and dominance scores within two positive and two negative emotion categories. The results showed no significant difference between amusement and tenderness (*t* = −0.51, *p* = 0.615) but a significant difference between anger and fear on the liking dimension (*t* = −2.31, *p* = 0.027). There was no significant difference between amusement and tenderness (*t* = −0.99, *p* = 0.330) but a significant difference between anger and fear on the familiarity dimension (*t* = 3.78, *p* = 0.001). No significant differences between amusement and tenderness (*t* = −1.78, *p* = 0.083) as well as anger and fear (*t* = 0.76, *p* = 0.454) were found.

### Gender Differences in Emotional EEG Response

Gender differences were reported in a previous study on physiological responses elicited by film clips (Fernández et al., 2012). EEG asymmetries were computed as power at the right hemisphere minus power at the left hemisphere. For example, F3/F4 theta asymmetry was computed as theta power at the F4 site minus theta power at the F3 site. Thus, 28 independent sample *t* tests were performed on the theta power of four emotion categories to test gender differences at FP1/FP2 asymmetry, F3/F4 asymmetry, F_Z_, FC_Z_, C_Z_, CP_Z_ and P_Z_ sites. The results showed that only men had significantly higher theta power at the F_Z_ site for the amusing film (*t*_(31)_ = 2.22, *p* = 0.034, *d* = 0.22) than for the tender film (*t*_(31)_ = 2.15, *p* = 0.040, *d* = 0.75). Similarly, 28 independent sample *t* tests were performed on the alpha power of four emotion categories to test gender differences at FP1/FP2 asymmetry, F3/F4 asymmetry, F_Z_, FC_Z_, C_Z_, CP_Z_ and P_Z_ sites. Twenty independent sample *t* tests were performed on the beta power of four emotion categories at the F_Z_, FC_Z_, C_Z_, CP_Z_ and P_Z_ sites. No significant differences between women and men were found.

### Frontal Asymmetry of Two Positive Emotions and Two Negative Emotions

#### FP1/FP2 Site

##### Comparison Between Tenderness and Amusement

Two-way repeated measures MANOVA was conducted for the average power spectra of theta and alpha bands, with emotion category (amusement and tenderness) and hemisphere (left and right) as two within-subject variables at the FP1/FP2 site. The degrees of freedom were Greenhouse-Geisser corrected. Using Wilks’s statistic, there was a significant emotion category × hemisphere interaction effect on theta and alpha power at the FP1/FP2 site (*λ* = 0.679, *F*_(2,31)_ = 7.32, *p* = 0.002, $\eta_{\text{p}}^{2}$ = 0.321). Moreover, the main effects of both the emotion category (*λ* = 0.805, *F*_(2,31)_ = 3.75, *p* = 0.035, $\eta_{\text{p}}^{2}$ = 0.195) and the hemisphere (*λ* = 0.437, *F*_(2,31)_ = 19.94, *p* < 0.001, $\eta_{\text{p}}^{2}$ = 0.563) on theta and alpha power at the FP1/FP2 site were significant. The means and standard deviations for the mean power values of the theta band are presented in Table 2.

**Table 2:** Means and standard deviations for the mean power values of the theta and alpha bands [M (SD)].

			Amusement	Tenderness	Anger	Fear
theta	FP1/FP2	Left (FP1)	0.54 (0.26)	0.58 (0.32)	0.50 (0.29)	0.55 (0.29)
		Right (FP2)	0.88 (0.39)	1.06 (0.46)	0.76 (0.38)	0.91 (0.49)
	F3/F4	Left (F3)	1.00 (0.34)	1.01 (0.34)	0.85 (0.32)	0.99 (0.41)
		Right (F4)	1.30 (0.37)	1.42 (0.41)	1.10 (0.35)	1.28 (0.42)
	F_left_/F_right_	Left (F_left_)	0.77 (0.27)	0.80 (0.29)	0.67 (0.25)	0.77 (0.30)
		Right (F_right_)	1.09 (0.32)	1.24 (0.35)	0.93 (0.28)	1.10 (0.34)
alpha	FP1/FP2	Left (FP1)	0.41 (0.27)	0.45 (0.31)	0.47 (0.41)	0.47 (0.43)
		Right (FP2)	0.54 (0.28)	0.62 (0.25)	0.57 (0.37)	0.60 (0.36)
	F3/F4	Left (F3)	0.62 (0.32)	0.70 (0.29)	0.64 (0.32)	0.64 (0.38)
		Right (F4)	0.77 (0.31)	0.90 (0.32)	0.74 (0.33)	0.83 (0.43)
	F_left_/F_right_	Left (F_left_)	0.52 (0.26)	0.58 (0.26)	0.55 (0.31)	0.55 (0.36)
		Right (F_right_)	0.65 (0.26)	0.76 (0.26)	0.66 (0.32)	0.71 (0.35)

Then, separate repeated measures univariate analyses of variances (ANOVAs) on the outcome variables revealed a significant emotion category × hemisphere interaction effect on theta power at the FP1/FP2 site (*F*_(1,32)_ = 15.02, *p* < 0.001, $\eta_{\text{p}}^{2}$ = 0.319). For all ANOVAs, the degrees of freedom were Greenhouse-Geisser corrected where appropriate. Next, simple effect analyses with a Bonferroni correction revealed no significant theta power value at FP1 between tenderness and amusement (*p* = 0.031). However, the theta power value of tenderness was significantly higher than that of amusement at FP2 (*p* = 0.002).

A nonsignificant category × hemisphere interaction effect on alpha power was found (*F*_(1,32)_ = 2.76, *p* = 0.106, $\eta_{\text{p}}^{2}$ = 0.079). However, the main effects of emotion category (*F*_(1,32)_ = 4.33, *p* = 0.015, $\eta_{\text{p}}^{2}$ = 0.170) and hemisphere (*F*_(1,32)_ = 13.21, *p* < 0.001, $\eta_{\text{p}}^{2}$ = 0.292) were significant. *Post hoc* analyses showed that the alpha power of tenderness was significantly higher than that of amusement (*p* = 0.046). Additionally, the alpha power at FP2 was significantly higher than that at FP1 (*p* = 0.001).

##### Comparison Between Anger and Fear

Two-way repeated measures MANOVA was conducted for the average power spectra of theta and alpha bands, with emotion category (anger and fear) and hemisphere (left and right) as two within-subject variables at the FP1/FP2 site. Using Wilks’s statistic, there was a significant emotion category × hemisphere interaction effect on theta and alpha power at the FP1/FP2 site (*λ* = 0.782, *F*_(2,31)_ = 4.32, *p* = 0.022, $\eta_{\text{p}}^{2}$ = 0.218). The main effects of both emotion category (*λ* = 0.796, *F*_(2,31)_ = 3.96, *p* = 0.029, $\eta_{\text{p}}^{2}$ = 0.204) and hemisphere (*λ* = 0.566, *F*_(2,31)_ = 11.87, *p* < 0.001, $\eta_{\text{p}}^{2}$ = 0.434) on theta and alpha power at the FP1/FP2 site were also significant. The means and standard deviations for the mean power values of the theta band are presented in Table 2.

Similarly, separate repeated measures univariate ANOVAs on the outcome variables revealed a significant emotion category × hemisphere interaction effect on theta power at the FP1/FP2 site (*F*_(1,32)_ = 7.86, *p* = 0.009, $\eta_{\text{p}}^{2}$ = 0.197). Simple effect analyses with a Bonferroni correction revealed no significant difference between anger and fear on theta power at FP1 (*p* = 0.147) but a significant difference at FP2 (*p* = 0.003).

For alpha power, no significant emotion category × hemisphere interaction effect was found (*F*_(1,32)_ = 1.74, *p* = 0.197, $\eta_{\text{p}}^{2}$ = 0.052). No main effect of emotion category (*F*_(1,32)_ = 0.08, *p* = 0.785, $\eta_{\text{p}}^{2}$ = 0.002) was found. The main effect of hemisphere (*F*_(1,32)_ = 3.35, *p* = 0.077, $\eta_{\text{p}}^{2}$ = 0.095) was marginally significant.

#### F3/F4 Site

##### Comparison Between Tenderness and Amusement

Similar two-way repeated measures MANOVA was conducted at the F3/F4 site. Using Wilks’s statistic, there was a significant emotion category × hemisphere interaction effect on theta and alpha power at the F3/F4 site (*λ* = 0.690, *F*_(2,31)_ = 6.96, *p* = 0.003, $\eta_{\text{p}}^{2}$ = 0.310). The main effects of both emotion category (*λ* = 0.813, *F*_(2,31)_ = 3.56, *p* = 0.041, $\eta_{\text{p}}^{2}$ = 0.187) and hemisphere (*λ* = 0.181, *F*_(2,31)_ = 69.95, *p* < 0.001, $\eta_{\text{p}}^{2}$ = 0.819) on theta and alpha power at the FP1/FP2 site were also significant.

Then, separate repeated measures univariate ANOVAs on the outcome variables revealed a significant emotion category × hemisphere interaction effect on theta power at the F3/F4 site (*F*_(1,32)_ = 8.30, *p* = 0.007, $\eta_{\text{p}}^{2}$ = 0.206). The following simple effect analysis showed no significant difference between tenderness and amusement on the average power of the theta band both at F3 (*p* = 0.795) and F4 (*p* = 0.094). However, based on the trend, the theta power of tenderness is higher than that of amusement at F4.

Furthermore, a significant emotion category × hemisphere interaction effect on alpha power at the F3/F4 site (*F*_(1,32)_ = 9.80, *p* = 0.004, $\eta_{\text{p}}^{2}$ = 0.234) was found. The follow-up simple effect analyses revealed significant differences between tenderness and amusement on the average power of the theta band at F3 (*p* = 0.05) and F4 (*p* = 0.004).

##### Comparison Between Anger and Fear

Using Wilks’s statistic, there was a significant emotion category × hemisphere interaction effect on theta and alpha power at the F3/F4 site (*λ* = 0.633, *F*_(2,31)_ = 8.98, *p* = 0.001, $\eta_{\text{p}}^{2}$ = 0.367). The main effect of hemisphere (*λ* = 0.180, *F*_(2,31)_ = 70.53, *p* < 0.001, $\eta_{\text{p}}^{2}$ = 0.820) on theta and alpha power at the FP1/FP2 site was also significant.

Then, separate repeated measures univariate ANOVAs on the outcome variables revealed a marginally significant emotion category × hemisphere interaction effect on theta power at the F3/F4 site (*F*_(1,32)_ = 3.70, *p* = 0.063, $\eta_{\text{p}}^{2}$ = 0.104). Simple effect analyses found significant differences between anger and fear on theta power at F3 (*p* = 0.041) and F4 (*p* = 0.016). A significant emotion category × hemisphere interaction effect on alpha power was found (*F*_(1,32)_ = 10.88, *p* = 0.002, $\eta_{\text{p}}^{2}$ = 0.254). Simple effect analyses found no significant differences between anger and fear on theta power at F3 (*p* = 0.945) and F4 (*p* = 0.233). However, a statistical trend could be observed; the alpha power of fear was higher than that of anger at F4.

#### F_left_/F_right_ Site

##### Comparison Between Tenderness and Amusement

F_left_/F_right_ was the average power over the left and right electrodes, respectively (F_left_ = (FP1 + F3)/2, F_right_ = (FP2 + F4)/2). Two-way repeated measures MANOVA was also conducted at this pair of sites. Using Wilks’s statistic, there was a significant emotion category × hemisphere interaction effect on theta and alpha power at the F_left_/F_right_ site (*λ* = 0.649, *F*_(2,31)_ = 8.39, *p* = 0.001, $\eta_{\text{p}}^{2}$ = 0.351). The main effects of both emotion category (*λ* = 0.820, *F*_(2,31)_ = 3.40, *p* = 0.046, $\eta_{\text{p}}^{2}$ = 0.180) and hemisphere (*λ* = 0.208, *F*_(2,31)_ = 59.03, *p* < 0.001, $\eta_{\text{p}}^{2}$ = 0.792) on theta and alpha power at the FP1/FP2 site were also significant.

**Figure 3:**
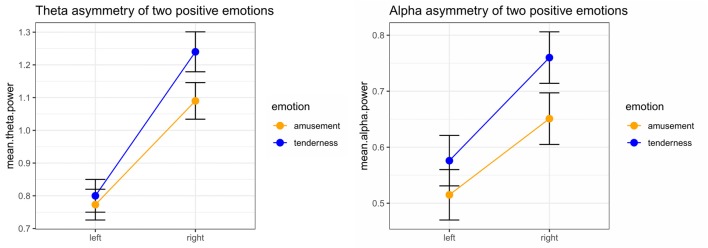
Significant emotion category × hemisphere interactions for the mean power values of theta and the theta band at the F_left_/F_right_ site between amusement and tenderness.

Significant emotion category × hemisphere interaction effects on both theta power (*F*_(1,32)_ = 15.05, *p* < 0.001, $\eta_{\text{p}}^{2}$ = 0.320) and alpha power (*F*_(1,32)_ = 7.13, *p* = 0.012, $\eta_{\text{p}}^{2}$ = 0.182) were found. For theta power, simple effect analysis found no significant difference between the tender movie and amusing movie (*p* = 0.510) at F_left_. In contrast, the tender movie showed significantly higher theta power at F_right_ than did the amusing movie (*p* = 0.015). Regarding alpha power, the difference between tenderness and amusement at F_left_ was marginally significant (*p* = 0.061) and significant at F_right_ (*p* = 0.007). The results of the interaction effect are shown in Figure 3.

##### Comparison Between Anger and Fear

Two-way repeated measures MANOVA was conducted at the F_left_/F_right_ site. Using Wilks’s statistic, there was a significant emotion category × hemisphere interaction effect on theta and alpha power at the F_left_/F_right_ site (*λ* = 0.603, *F*_(2,31)_ = 10.19, *p* < 0.001, $\eta_{\text{p}}^{2}$ = 0.397). The main effects of both emotion category (*λ* = 0.824, *F*_(2,31)_ = 3.31, *p* = 0.050, $\eta_{\text{p}}^{2}$ = 0.176) and hemisphere (*λ* = 0.309, *F*_(2,31)_ = 34.73, *p* < 0.001, $\eta_{\text{p}}^{2}$ = 0.691) on theta and alpha power at the FP1/FP2 site were also significant.

**Figure 4:**
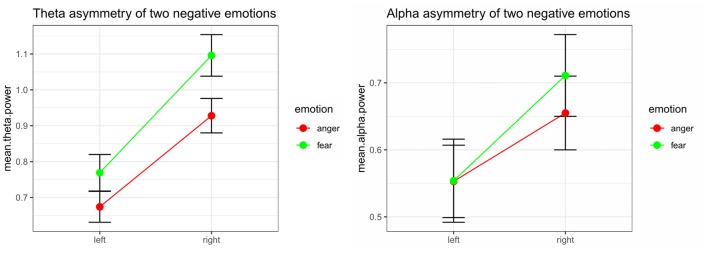
Significant emotion category × hemisphere interactions for the mean power values of theta and the theta band at the F_left_/F_right_ site between anger and fear.

Significant emotion category × hemisphere interaction effects on both theta power (*F*_(1,32)_ = 8.48, *p* = 0.006, $\eta_{\text{p}}^{2}$ = 0.210) and alpha power (*F*_(1,32)_ = 10.52, *p* = 0.003, $\eta_{\text{p}}^{2}$ = 0.247) were found. For theta power, simple effect analysis found a marginally significant difference between the tender movie and amusing movie (*p* = 0.052) at F_left_ and a significant difference at F_right_ (*p* = 0.006). Regarding alpha power, the difference between tenderness and amusement at both F_left_ (*p* = 0.961) and F_right_ (*p* = 0.331) were nonsignificant. The results of the interaction effect are shown in Figure 4.

### Midline Power of Three Bands Between Two Positive and Two Negative Emotions

Paired-samples *t* tests were conducted for the average power of theta, alpha and beta bands between two positive emotions and two negative emotions (F_Z_, FC_Z_, C_Z_, CP_Z_ and P_Z_ electrodes were examined). The means, standard deviations and *t* values for the midline power values of the three bands are shown in Table 3. Paired-samples *t* test of midline theta power between amusement and tenderness showed no significant difference between amusement and tenderness. However, the mean power values of the theta band during the scary movie were significantly higher than those during the angry movie at the F_Z_ (*t*_(32)_ = −2.64, *p* = 0.013, *d* = 0.40), FC_Z_ (*t*_(32)_ = −2.65, *p* = 0.012, *d* = 0.46), and C_Z_ (*t*_(32)_ = −2.32, *p* = 0.027, *d* = 0.42) sites and marginally significant at the CP_Z_ (*t*_(32)_ = −1.99, *p* = 0.056, *d* = 0.39) site.

**Table 3:** The means, standard deviations, and *t* values for the midline power values of three bands.

	Theta		Alpha		Beta	
	Anger–Fear M (SD)	*t*	Amusement–Tenderness M (SD)	*t*	Amusement–Tenderness M (SD)	*t*
F_Z_	−0.23 (0.49)	−2.64	−0.12 (0.27)	−2.58	−0.10 (0.25)	−2.42
FC_Z_	−0.27 (0.59)	−2.65	−0.14 (0.31)	−2.53	−0.12 (0.25)	−2.86
C_Z_	−0.22 (0.54)	−2.32	−0.16 (0.36)	−2.57	−0.11 (0.27)	−2.41
CP_Z_	−0.18 (0.52)	−1.99	−0.16 (0.40)	−2.27	−0.14 (0.25)	−3.20
P_Z_	−0.13 (0.52)	−1.42	−0.17 (0.41)	−2.41	−0.17 (0.28)	−3.49

Conversely, for the alpha band, there were no significant differences between the angry and scary movies at all midline sites, whereas the mean power values of the alpha band during the tender movie were significantly higher than those during the amusing movie at the F_Z_ (*t*_(32)_ = −2.58, *p* = 0.015, *d* = 0.32), FC_Z_ (*t*_(32)_ = −2.53, *p* = 0.017, *d* = 0.32), C_Z_ (*t*_(32)_ = −2.57, *p* = 0.015, *d* = 0.40), CP_Z_ (*t*_(32)_ = −2.27, *p* = 0.030, *d* = 0.36) and P_Z_ (*t*_(32)_ = −2.41, *p* = 0.022, *d* = 0.41) sites.

Similarly, as shown by the paired-samples *t* test, for the beta band, there were no significant differences between the angry and scary movies at all midline sites. In addition, the mean power values of the beta band during the tender movie were significantly higher than those during the amusing movie at the F_Z_ (*t*_(32)_ = −2.42, *p* = 0.021, *d* = 0.32), FC_Z_ (*t*_(32)_ = −2.86, *p* = 0.007, *d* = 0.34), C_Z_ (*t*_(32)_ = −2.41, *p* = 0.022, *d* = 0.31), CP_Z_ (*t*_(32)_ = −3.20, *p* = 0.003, *d* = 0.41) and P_Z_ (*t*_(32)_ = −3.49, *p* = 0.001, *d* = 0.54) sites.

### Regression on the Subjective Rating of Two Positive Emotions and Two Negative Emotions

Several stepwise multiple regression analyses were performed to detect the significant EEG predictors of subjective ratings of four emotion categories. First, the rating score of amusement was selected to serve as the dependent variable. Alpha power and theta power at FP1/FP2 asymmetry, F3/F4 asymmetry and 5 midline sites (F_Z_, FC_Z_, C_Z_, CP_Z_, P_Z_) and beta power at the same 5 midline sites were selected as the independent variables. Only F3/F4 alpha asymmetry entered the stepwise multiple linear regression equation and accounted for a significant portion of the variance (*F*_(1,130)_ = 5.086, *p* = 0.026, adjusted *R*^2^ = 0.03). All regression coefficients are displayed in Table 4. Second, the rating score of tenderness was selected to serve as the dependent variable. Stepwise multiple linear regression analysis determined that FP1/FP2 theta asymmetry, F3/F4 alpha and theta asymmetry, and FC_Z_ beta power were the significant predictors of the subjective rating of tenderness (*F*_(4,127)_ = 7.133, *p* < 0.001, adjusted *R*^2^ = 0.158). Third, a similar stepwise multiple linear regression analysis with a rating score of anger as the dependent variable showed that F3/F4 theta asymmetry was the significant predictor (*F*_(1,130)_ = 9.075, *p* = 0.003, adjusted *R*^2^ = 0.058). Additionally, stepwise multiple linear regression analysis with a rating score of fear as the dependent variable showed that F3/F4 alpha asymmetry and F3/F4 theta asymmetry served as the significant predictors (*F*_(2,129)_ = 15.117, *p* < 0.001, adjusted *R*^2^ = 0.177).

**Table 4:** Stepwise multiple linear regression analysis for subjective ratings of four emotions.

	Predictor	Unstandardized		Standardized	*t*	*p*
		*B*	Std. Error	*β*		
Amusement	(Constant)	2.917	0.287			
	F3/F4 alpha asymmetry	−1.395	0.618	−0.194	−2.255	0.026
Tenderness	(Constant)	−0.526	0.815			
	FP1/FP2 theta asymmetry	1.095	0.552	0.160	1.982	0.050
	F3/F4 alpha asymmetry	−1.443	0.608	−0.195	−2.374	0.019
	F3/F4 theta asymmetry	4.390	1.198	0.301	3.663	<0.001
	FC_Z_ beta	1.984	0.694	0.231	2.858	0.005
Anger	(Constant)	3.355	0.319			
	F3/F4 theta asymmetry	−2.017	0.688	−0.255	−3.013	0.003
Fear	(Constant)	3.399	0.463			
	F3/F4 alpha asymmetry	−3.785	1.249	−0.245	−3.03	0.003
	F3/F4 theta asymmetry	3.233	0.633	0.413	5.105	<0.001

### Partial Correlation Analysis

To explore how the significant EEG index reflects modulations of the subjective evaluations, we calculated partial correlations between EEG powers and subjective ratings. First, significant EEG measures were selected in which the power of one emotion category was significantly higher than that of the other. Partial correlation analysis was applied to the EEG measures of two positive emotions and subjective assessments in which arousal, valence and familiarity dimensions were controlled. The *p*-values were corrected according to the false discovery rate (FDR) using Benjamini and Hochberg’s (BH) method (Benjamini and Hochberg, 1995). A significant correlation was found between liking and F3/F4 theta asymmetry (*r* = 0.265, *p* = 0.034). Specifically, increased F3/F4 theta asymmetry (higher F4 theta power than F3) was correlated with higher ratings of liking. The same statistical trend was also found for liking and F_left_/F_right_ theta asymmetry; higher F_right_/F_left_ theta asymmetry showed a higher rating of liking (*r* = 0.303, *p* = 0.03).

Similar partial correlation analysis was successively applied to EEG measures of two negative emotions and subjective assessments. The *p*-values were corrected in the same way as above. Significant correlations were found between liking and FP1/FP2 theta asymmetry (*r* = 0.301, *p* = 0.024), liking and F_right_/F_left_ theta asymmetry (*r* = 0.324, *p* = 0.016), liking and F_Z_ theta power (*r* = − 0.273, *p* = 0.034), liking and FC_Z_ theta power (*r* = − 0.355, *p* = 0.011), liking and C_Z_ theta power (*r* = − 0.394, *p* = 0.004), and liking and CP_Z_ theta power (*r* = − 0.399, *p* = 0.004). Increased frontal theta asymmetry was associated with higher ratings of liking, whereas increased midline theta power was correlated with lower ratings of liking. Additionally, significant correlations were observed between dominance and FP1/FP2 theta asymmetry (*r* = − 0.257, *p* = 0.039) and dominance and F_right_/F_left_ theta asymmetry (*r* = − 0.270, *p* = 0.034). Moreover, lower frontal theta asymmetry was related to higher ratings of dominance. No other significant correlations were found for the alpha and beta bands.

### Classification of Two Positive Emotions and Two Negative Emotions Using EEG Signals

Three SVMs were built for classification. One SVM was for the classification of tenderness and amusement, another was for the classification of anger and fear, and the third was for the classification of all four emotions. Five sets of features were extracted. The first set was the original 75 EEG features extracted from three power bands (alpha, theta and beta) at 25 electrodes (FP1, FP2, F7, F3, F4, F8, FT7, FC3, FC4, FT8, T3, C3, C4, T4, TP7, CP3, CP4, TP8, T5, P3, PZ, P4, T6, O1, OZ and O2) without five midline sites that we focused on in the current study. The second set was composed of the original 75 EEG features as well as 15 midline features from three powers bands (alpha, theta and beta) at five midline sites (F_Z_, FC_Z_, C_Z_, CP_Z_ and P_Z_). The third set was composed of the original 75 EEG features and 4 asymmetry features (FP1/FP2 theta asymmetry, FP1/FP2 alpha asymmetry, F3/F4 theta asymmetry and F3/F4 alpha asymmetry). The fourth set was composed of the original 75 EEG features and 19 new features that were mentioned above. The last set was composed of the 19 new features only.

The results of classification using the SVM classifier are displayed in Table 5. For the binary classification, our binary models achieved 56.06% classification accuracy for tenderness and amusement and 57.58% for anger and fear in the first set. The new 15 midline features added in the second set achieved better classification accuracies for tenderness and amusement (75.76%) and for anger and fear (63.64%) than did those in the first set. Similarly, compared to those in the first set, 4 new asymmetry features added in the third set improved the accuracy up to 72.73% for tenderness and amusement and 75.76% for anger and fear. All 19 new features added in the fourth set obtained the best classification accuracies for the two positive emotions (77.27%) and two negative emotions (83.33%). The recognition of emotions using only 19 new features outperformed the recognition using original features. Regarding the classification among four emotion categories, compared with the other four feature sets, the last set containing all 19 new features obtained the best classification accuracy (66.67%). Thus, using 19 new features could improve the accuracy over that obtained using the original features.

**Table 5:** Classification results using electroencephalographic (EEG) signals as features and support vector machine (SVM) classifier.

Feature description	Classification accuracy with SVM classifier		
	Tenderness/amusement	Anger/fear	Four emotions
Original 75 features from three bands	0.5606	0.5758	0.3864
Original 75 features and 15 new midline features	0.7576	0.6364	0.5455
Original features and 4 new asymmetry features	0.7273	0.7576	0.5682
Original features and all 19 new features	0.7727	0.8333	0.6667
19 new features only	0.6452	0.7879	0.4848

## Discussion

The present study examined the EEG patterns that can distinguish two emotions that are similar in both arousal and valence dimensions. The following main points can synthesize the results of the present research. First, frontal theta asymmetry and alpha asymmetry were found for positive and negative emotions. Second, the results indicated that theta power at midline was significantly different between negative emotions, whereas alpha and beta power at midline sites were significantly different between positive emotions. Finally, we proved that liking was positively related to frontal theta asymmetry and negatively related to midline theta power, while dominance was negatively related to frontal theta asymmetry. The results of this study indicate that emotions similar in valence and arousal could be distinguished by frontal theta asymmetry, frontal alpha asymmetry, midline theta power, midline alpha power and midline beta power.

For positive emotion, greater frontal alpha asymmetry was observed in the tender movie than in the amusing movie. Compared with the amusing movie, the tender movie elicited significantly higher frontal alpha power in the right hemisphere than in the left hemisphere. A particularly interesting explanation for the relationship between frontal asymmetry and two positive emotions is the possible involvement of the motivational dimension of emotion. Motivation drive refers to the desire to approach a reward or escape a punishment (Davidson, 1995). More than 150 studies have examined the relationship between frontal asymmetry and various emotional and motivational states during the past three decades (Coan and Allen, 2004; Thibodeau et al., 2006). Notably, frontal alpha asymmetry is considered to reflect the valence dimension of emotions earlier (Poole and Gable, 2014). However, positive emotion often correlates with approach-related motivation with more left frontal brain activity, whereas negative emotion often correlates with withdrawal-related motivation with more right frontal brain activity (Harmon-Jones and Sigelman, 2001; Rohlfs and Ramírez, 2006). Due to the overlap between the valence and approach-withdrawal motivational direction, the valence hypothesis was largely subsumed in the approach-withdrawal model (Coan and Allen, 2004; Davidson, 2004).

According to the current results, tenderness showed significantly greater right than left frontal alpha power, whereas amusement did not show this discrepancy. This finding might indicate that tenderness is related to greater approach motivation than amusement. A previous study of the motivational dimension of emotion divided positive emotion into low-approach motivational positive emotion (such as peace and amusement) and high-approach motivational positive emotion (such as passion and aspiration; Gable and Harmonjones, 2010). In addition to the current results, tenderness can be regarded as a high-approach motivational positive emotion, whereas amusement is a low-approach motivational positive emotion. This result could be explained by the emotional induction stimuli of tenderness. Tenderness was evoked by the perception of vulnerability, which manifested the target as weak as well as defenseless and in need of protection (Dijker, 2010). Moreover, the experience of empathic emotions such as tenderness can further promote the intention to love, care, nurture and protect the offspring and young (Niedenthal et al., 2009). Compared with tenderness, amusement was considered a common positive emotion that was elicited by external stimuli, and it is impossible to inherently drive people toward something in the environment (Fredrickson and Branigan, 2005). Additionally, frontal theta asymmetry was significantly different between amusement and tenderness. The results also showed that frontal theta asymmetry was positively related to the liking dimension. According to the results of self-assessment ratings, tenderness was rated higher than amusement on liking but not significantly. Thus, frontal theta asymmetry may reflect a liking dimension, which seems to have something in common with approach motivation. This relationship must be verified in future research.

For negative emotion, there was greater frontal theta asymmetry during the scary movie than during the angry movie. Compared with the angry movie, the scary movie elicited significantly higher frontal theta power in the right hemisphere than in the left hemisphere. For the difference between two negative emotions, another dimension of emotion dominance is discussed as follows. Dominance was defined as the amount of influence we feel the environment has upon us and vice versa; it ranges from extreme feelings of lack of control or of being influenced by one’s surroundings to feelings of being powerful and fully in control (Osgood, 1966; Yani-de-Soriano and Foxall, 2006). Additionally, dominance refers to whether one feels in control, powerful, or overwhelmed (Broekens, 2012). Some researchers have found that left frontal arousal may be associated with a feeling of dominance and right frontal arousal with a feeling of submission (Demaree et al., 2005). Moreover, numerous earlier studies have shown that anger is associated with feelings of dominance, whereas fear and disgust are associated with feelings of submission (Russell and Mehrabian, 1977; Mehrabian, 1978, 1980, 1996). Although the result of self-assessments in the current study showed no significant difference between anger and fear in the dominance dimension due to different measurements, the rating scores in the movie database from which the clips were chosen showed that the angry movie was in fact rated more dominant than the scary movie. Therefore, as shown in the current study, higher theta power in the right hemisphere (fear) might be associated with lower ratings of dominance. Furthermore, the results from correlation analysis verified that frontal theta asymmetry was significantly related to dominance and that increased frontal theta asymmetry (higher right hemisphere power) was associated with a lower rating of dominance. Thus, the dominance dimension plays a major role in differentiating between anger and fear (Broekens and Degroot, 2004), and the difference between anger and fear in frontal theta activity may reflect the dominance dimension of emotion. A previous study confirmed that the beta/alpha activity ratio in the frontal lobe added to beta activity at the parietal lobe could reflect the dominance dimension of emotion (Bos, 2006). Additionally, the results of the current study show that the dominance dimension of emotion was related to theta activity in the frontal lobe when subjects experienced negative emotions. However, the relationship between frontal theta activity and dominance remains to be tested by further studies.

For frontal alpha asymmetry between anger and fear, an explanation for motivational direction can be noted, as mentioned above. Numerous studies have reported that anger commonly evokes behavioral tendencies of approach and is associated with approach motivation, and fear as a typical negatively valence emotion is linked to withdrawal motivation (Harmon-Jones and Allen, 1998; Harmon-Jones et al., 2008; He et al., 2010; Wilkowski and Meier, 2010; Kelley and Schmeichel, 2014). However, both anger and fear induced by movie clips showed significantly higher alpha power in the right hemisphere than in the left hemisphere, which shows that both the negative emotions might display approach-related motivation. This finding is somewhat different from previous results regarding the fearful emotion due to different measurements. Based on empirical evidence, participants may be interested in the plot of a scary movie and are curious about what will occur next even though they are experiencing fear. The lack of assessments of motivational direction in rating scales after watching movie clips was a limitation of the current study.

Furthermore, the results indicated that the scary movie elicited significantly greater theta power at midline sites than did the angry movie, whereas the tender movie elicited significantly greater alpha and beta power at midline sites than the amusing movie. Very few previous emotional models and theories offer straightforward hypotheses for the difference between emotion categories in midline power. For example, midline theta and alpha activity was related to positive emotional experience (Aftanas and Golocheikine, 2001). Current results revealed that midline theta power was also related to negative emotions such as anger and fear. In addition, evidence from a meta-analysis argued that frontal midline theta reflected anxiety and a plausible mechanism for optimally adjusting behavior to uncertainty (Cavanagh and Shackman, 2015). Moreover, frontal midline theta was a specific indicator of attention engagement (Kao et al., 2013) and was modulated by anticipatory situational threat (Osinsky et al., 2017). It can be inferred that participants might experience more anxiety during a scary movie clip than during an angry movie clip. Participants must concentrate their attention on the anticipatory threat in the context of the movie to avoid being scared by the scene. In regard to positive emotions, notably, midline alpha as well as beta power can be used to distinguish amusement from tenderness.

The results of stepwise multiple linear regressions and nonlinear binary classification proved the ability of EEG data to distinguish emotions in a similar valence-arousal space. The regression results proved that F3/F4 alpha asymmetry could negatively predict subjective ratings of both tenderness and amusement. Additionally, theta asymmetry at FP1/FP2 and F3/F4 sites could significantly predict subjective ratings of tenderness, which is in accordance with the significant theta asymmetry differences between tenderness and amusement observed at these sites. For two negative emotions, evidence indicated that F3/F4 theta asymmetry could negatively predict subjective ratings of anger but positively predict fear. Additionally, F3/F4 alpha asymmetry could negatively predict subjective ratings of fear. These results mainly indicated that frontal alpha and theta asymmetry could predict subjective feelings of two positive and two negative emotions in different patterns. Binary classification using the SVM classifier also demonstrated the effectiveness of using EEG frontal asymmetry and midline power as features to recognize different emotions that are similar in valence and arousal. Notably, a previous study successfully distinguished positive and negative affective states using EEG power from all channels across the scalp (Nie et al., 2011; Stikic et al., 2014). These features (without midline and asymmetry features) were selected as the original feature set in the current study to classify two emotions that are similar in valence, which gained above-chance level accuracy. After adding new features that were proposed in the current study to the original features, the recognition accuracy for binary classification improved approximately 20%. Moreover, using midline and asymmetry features improved the classification accuracy compared to using the original features. It is also worth mentioning that the current classification accuracy for two negative emotions using new features outperformed only a previous study with an accuracy of 66.3% using EEG asymmetry as well as coherences to classify film-induced anger and fear emotion (Park et al., 2013).

In short, the results of the current study indicate that activity in theta, alpha and beta bands can differentiate emotions that are similar in valence and arousal. Frontal theta asymmetry, frontal alpha asymmetry, midline theta power and midline beta power were effective in distinguishing amusement and tenderness, anger and fear. The results are well suited for emotion recognition and provide evidence that theta, alpha and beta powers play an important role in emotion processing. Future studies might concentrate on the differences among more basic emotions and more complex emotions in brain activities. In addition, the relationships between these physiological differences and emotional dimensions, such as dominance and motivation, must be further explored.

## Author Contributions

GZ and YG designed the experiments and wrote the manuscript. YZ carried out the experiments, analyzed experimental data and wrote the manuscript.

## Conflict of Interest Statement

The authors declare that the research was conducted in the absence of any commercial or financial relationships that could be construed as a potential conflict of interest.

## References

[B1] Aftanas L. I. Golocheikine S. A. (2001). Human anterior and frontal midline theta and lower α reflect emotionally positive state and internalized attention: high-resolution EEG investigation of meditation. Neurosci. Lett. 310, 57–60. 10.1016/s0304-3940(01)02094-8 11524157

[B2] Aftanas L. I. Reva N. V. Savotina L. N. Makhnev V. P. (2006). Neurophysiological correlates of induced discrete emotions in humans: an individually oriented analysis. Neurosci. Behav. Physiol. 36, 119–130. 10.1007/s11055-005-0170-6 16380825

[B3] Allen J. J. Coan J. A. Nazarian M. (2004). Issues and assumptions on the road from raw signals to metrics of frontal EEG asymmetry in emotion. Biol. Psychol. 67, 183–218. 10.1016/j.biopsycho.2004.03.007 15130531

[B4] Balconi M. Mazza G. (2010). Lateralisation effect in comprehension of emotional facial expression: a comparison between EEG alpha band power and behavioural inhibition (BIS) and activation (BAS) systems. Laterality 15, 361–384. 10.1080/13576500902886056 19536685

[B5] Balconi M. Pozzoli U. (2009). Arousal effect on emotional face comprehension: frequency band changes in different time intervals. Physiol. Behav. 97, 455–462. 10.1016/j.physbeh.2009.03.023 19341748

[B6] Barrett L. F. (2011). Was Darwin wrong about emotional expressions? Curr. Dir. Psychol. Sci. 20, 400–406. 10.1177/0963721411429125

[B7] Barrett L. F. (2012). Emotions are real. Emotion 12, 413–429. 10.1037/a0027555 22642358

[B8] Barrett L. F. Mesquita B. Ochsner K. N. Gross J. J. (2007). The experience of emotion. Annu. Rev. Psychol. 58, 373–403. 10.1146/annurev.psych.58.110405.085709 17002554 PMC1934613

[B9] Bekkedal M. Y. Rossi J. III. Panksepp J. (2011). Human brain EEG indices of emotions: delineating responses to affective vocalizations by measuring frontal theta event-related synchronization. Neurosci. Biobehav. Rev. 35, 1959–1970. 10.1016/j.neubiorev.2011.05.001 21596060

[B10] Benjamini Y. Hochberg Y. (1995). Controlling the false discovery rate—a practical and powerful approach to multiple testing. J. R. Stat. Soc. 57, 289–300.

[B11] Bos D. O. (2006). EEG-based emotion recognition. The Influence of Visual and Auditory Stimuli. 56, 1–17.

[B13] Bradley M. M. Codispoti M. Cuthbert B. N. Lang P. J. (2001). Emotion and motivation I: defensive and appetitive reactions in picture processing. Emotion 1, 276–298. 10.1037/1528-3542.1.3.276 12934687

[B12] Bradley M. M. Lang P. J. (1994). Measuring emotion: the self-assessment manikin and the semantic differential. J. Behav. Ther. Exp. Psychiatry 25, 49–59. 10.1016/0005-7916(94)90063-9 7962581

[B14] Briesemeister B. B. Tamm S. Heine A. Jacobs A. M. (2013). Approach the good, withdraw from the bad—a review on frontal α asymmetry measures in applied psychological research. Psychology 4, 261–267. 10.4236/psych.2013.43a039

[B15] Broekens J. (2012). In defense of dominance. Int. J. Synt. Emot. 3, 33–42. 10.4018/jse.2012010103

[B16] Broekens J. Degroot D. (2004). “Scalable and flexible appraisal models for virtual agents,” in Proceedings of the 5th Game-On International Conference: Computer Games: Artificial Intelligence, Design and Education. (Ghent, Belgium), 208–215.

[B17] Cavanagh J. F. Shackman A. J. (2015). Frontal midline theta reflects anxiety and cognitive control: meta-analytic evidence. J. Physiol. Paris 109, 3–15. 10.1016/j.jphysparis.2014.04.003 24787485 PMC4213310

[B18] Coan J. A. Allen J. J. B. (2004). Frontal EEG asymmetry as a moderator and mediator of emotion. Biol. Psychol. 67, 7–49. 10.1016/j.biopsycho.2004.03.002 15130524

[B19] Coan J. A. Allen J. J. B. Harmon-Jones E. (2001). Voluntary facial expression and hemispheric asymmetry over the frontal cortex. Psychophysiology 38, 912–925. 10.1111/1469-8986.3860912 12240668

[B20] Darwin C. Prodger P. (1998). The Expression of the Emotions in Man and Animals. New York, NY: Oxford University Press.

[B21] Davidson R. J. (1995). Cerebral asymmetry, emotion and affective style. Mass. Inst. Technol. 12, 361–387.

[B22] Davidson R. J. (2004). What does the prefrontal cortex “do” in affect: perspectives on frontal EEG asymmetry research. Biol. Psychol. 67, 219–233. 10.1016/j.biopsycho.2004.03.008 15130532

[B23] Davidson R. J. Henriques J. (2000). “Regional brain function in sadness and depression,” in The Neuropsychology of Emotion, ed. Borod J. C. (New York, NY: Oxford University Press), 269–297.

[B24] Demaree H. A. Everhart D. E. Youngstrom E. A. Harrison D. W. (2005). Brain lateralization of emotional processing: historical roots and a future incorporating “dominance”. Behav. Cogn. Neurosci. Rev. 4, 3–20. 10.1177/1534582305276837 15886400

[B25] Depue R. A. Morrone-Strupinsky J. V. (2005). A neurobehavioral model of affiliative bonding: implications for conceptualizing a human trait of affiliation. Behav. Brain Sci. 28, 313–350. 10.1017/s0140525x05000063 16209725

[B26] Diaz A. Bell M. A. (2012). Frontal EEG asymmetry and fear reactivity in different contexts at 10 months. Dev. Psychobiol. 54, 536–545. 10.1002/dev.20612 22006522 PMC3571107

[B27] Dijker A. J. M. (2010). Perceived vulnerability as a common basis of moral emotions. Br. J. Soc. Psychol. 49, 415–423. 10.1348/014466609x482668 20030963

[B28] Ekman P. Cordaro D. (2011). What is meant by calling emotions basic. Emot. Rev. 3, 364–370. 10.1177/1754073911410740

[B29] Ellsworth P. C. Scherer K. R. (2003). “Appraisal processes in emotion,” in Series in Affective Science: Handbook of Affective Sciences, eds Davidson R. J. Scherer K. R. Goldsmith H. H. (New York, NY: Oxford University Press), 572–595.

[B30] Fernández C. Pascual J. C. Soler J. Elices M. Portella M. J. Fernández-Abascal E. (2012). Physiological responses induced by emotion-eliciting films. Appl. Psychophysiol. Biofeedback 37, 73–79. 10.1007/s10484-012-9180-7 22311202

[B31] Fontaine J. R. Scherer K. R. Roesch E. B. Ellsworth P. C. (2007). The world of emotions is not two-dimensional. Psychol. Sci. 18, 1050–1057. 10.1111/j.1467-9280.2007.02024.x 18031411

[B32] Fredrickson B. L. Branigan C. (2005). Positive emotions broaden the scope of attention and thought-action repertoires. Cogn. Emot. 19, 313–332. 10.1080/02699930441000238 21852891 PMC3156609

[B33] Gable P. Harmonjones E. (2010). The motivational dimensional model of affect: implications for breadth of attention, memory, and cognitive categorisation. Cogn. Emot. 24, 322–337. 10.1080/02699930903378305

[B34] Gross J. J. Levenson R. W. (1995). Emotion elicitation using films. Cogn. Emot. 9, 87–108. 10.1080/02699939508408966

[B35] Hamann S. (2012). Mapping discrete and dimensional emotions onto the brain: controversies and consensus. Trends Cogn. Sci. 16, 458–466. 10.1016/j.tics.2012.07.006 22890089

[B37] Harmon-Jones E. (2007). Trait anger predicts relative left frontal cortical activation to anger-inducing stimuli. Int. J. Psychophysiol. 66, 154–160. 10.1016/j.ijpsycho.2007.03.020 17561297

[B38] Harmon-Jones E. Allen J. J. B. (1998). Anger and frontal brain activity: EEG asymmetry consistent with approach motivation despite negative affective valence. J. Pers. Soc. Psychol. 74, 1310–1316. 10.1037/0022-3514.74.5.1310 9599445

[B36] Harmon-Jones E. Peterson C. Gable P. A. Harmon-Jones C. (2008). Anger and approach-avoidance motivation. Handbook of Approach and Avoidance Motivation, ed. Elliot A. J. (New York, NY: Psychology Press), 399–413.

[B39] Harmon-Jones E. Sigelman J. (2001). State anger and prefrontal brain activity: evidence that insult-related relative left-prefrontal activation is associated with experienced anger and aggression. J. Pers. Soc. Psychol. 80, 797–803. 10.1037/0022-3514.80.5.797 11374750

[B40] He J. Degnan K. A. McDermott J. M. Henderson H. A. Xu Q. Fox N. A. (2010). Anger and approach motivation in infancy: relations to early childhood inhibitory control and behavior problems. Infancy 15, 246–269. 10.1111/j.1532-7078.2009.00017.x 25705134 PMC4334138

[B41] Hu X. Yu J. Song M. Yu C. Wang F. Sun P.. (2017). EEG correlates of ten positive emotions. Front. Hum. Neurosci. 11:26. 10.3389/fnhum.2017.00026 28184194 PMC5266691

[B42] Javela J. J. Mercadillo R. E. Martín R. J. (2008). Anger and associated experiences of sadness, fear, valence, arousal, and dominance evoked by visual scenes. Psychol. Rep. 103, 663–681. 10.2466/pr0.103.7.663-681 19320198

[B43] Kalawski J. P. (2010). Is tenderness a basic emotion? Motiv. Emot. 34, 158–167. 10.1007/s11031-010-9164-y

[B81] Kao S. C. Huang C. J. Tsungmin H. (2013). Frontal midline theta is a specific indicator of optimal attentional engagement during skilled putting performance. J. Sport Exerc. Psychol. 35, 470–478. 10.1123/jsep.35.5.470 24197715

[B44] Kelley N. J. Schmeichel B. J. (2014). The effects of negative emotions on sensory perception: fear but not anger decreases tactile sensitivity. Front. Psychol. 5:942. 10.3389/fpsyg.2014.00942 25202299 PMC4141522

[B45] Kipp M. Martin J. C. (2009). “Gesture and emotion: can basic gestural form features discriminate emotions?,” in Proceedings of the 3rd International Conference on Affective Computing and Intelligent Interaction and Workshops. (Amsterdam, Netherlands), 1–8.

[B46] Kline J. P. Blackhart G. C. Woodward K. M. Williams S. R. Schwartz G. E. R. (2000). Anterior electroencephalographic asymmetry changes in elderly women in response to a pleasant and an unpleasant odor. Biol. Psychol. 52, 241–250. 10.1016/s0301-0511(99)00046-0 10725566

[B47] Koelstra S. Muhl C. Soleymani M. Jong-Seok L. Yazdani A. Ebrahimi T. (2012). DEAP: a database for emotion analysis using physiological signals. IEEE Trans. Affect. Comput. 3, 18–31. 10.1109/t-affc.2011.15

[B48] Kop W. J. Synowski S. J. Newell M. E. Schmidt L. A. Waldstein S. R. Fox N. A. (2011). Autonomic nervous system reactivity to positive and negative mood induction: the role of acute psychological responses and frontal electrocortical activity. Biol. Psychol. 86, 230–238. 10.1016/j.biopsycho.2010.12.003 21182891 PMC3061260

[B49] Labar K. S. LeDoux J. E. (1996). Partial disruption of fear conditioning in rats with unilateral amygdala damage: correspondence with unilateral temporal lobectomy in humans. Behav. Neurosci. 110, 991–997. 10.1037/0735-7044.110.5.991 8919001

[B50] Lang P. J. Greenwald M. K. Bradley M. M. Hamm A. O. (1993). Looking at pictures: affective, facial, visceral, and behavioral reactions. Psychophysiology 30, 261–273. 10.1111/j.1469-8986.1993.tb03352.x 8497555

[B51] Lindquist K. A. Wager T. D. Kober H. Bliss-Moreau E. Barrett L. F. (2012). The brain basis of emotion: a meta-analytic review. Behav. Brain Sci. 35, 121–143. 10.1017/s0140525x11000446 22617651 PMC4329228

[B52] Liu L. Yang M. Han Z. Zhou R. L. Cui H. (2014). Frontal EEG lateralization predicts individuals’ emotional flexibility. Sci. Sin. Vitae 44, 614–622. 10.1360/N052013-00061

[B53] Liu Y.-J. Yu M. Zhao G. Song J. Ge Y. Shi Y. (2017). “Real-time movie-induced discrete emotion recognition from EEG signals,” in Proceedings of the IEEE Transactions on Affective Computing (New York, NY: IEEE). 10.1109/TAFFC.2017.2660485

[B54] Mao M. (2013). Psychologically and Physiologically Measuring Music-induced Emotion and Its Application in Service Design. Tsinghua University: Beijing.

[B55] Mauss I. B. Robinson M. D. (2009). Measures of emotion: a review. Cogn. Emot. 23, 209–237. 10.1080/02699930802204677 19809584 PMC2756702

[B56] McFarland D. J. Parvaz M. A. Sarnacki W. A. Goldstein R. Z. Wolpaw J. R. (2016). Prediction of subjective ratings of emotional pictures by EEG features. J. Neural Eng. 14:016009. 10.1088/1741-2552/14/1/016009 27934776 PMC5476954

[B57] Mehrabian A. (1978). Measures of individual differences in temperament. Educ. Psychol. Meas. 38, 1105–1117. 10.1177/001316447803800431

[B58] Mehrabian A. (1980). Basic Dimensions for a General Psychological Theory. Cambridge, MA: Oelgeschlager, Gunn & Hain.

[B59] Mehrabian A. (1996). Pleasure-arousal-dominance: a general framework for describing and measuring individual differences in Temperament. Curr. Psychol. 14, 261–292. 10.1007/bf02686918

[B60] Mitchell D. J. McNaughton N. Flanagan D. Kirk I. J. (2008). Frontal-midline theta from the perspective of hippocampal “theta”. Prog. Neurobiol. 86, 156–185. 10.1016/j.pneurobio.2008.09.005 18824212

[B61] Mulligan K. Scherer K. R. (2012). Toward a working definition of emotion. Emot. Rev. 4, 345–357. 10.1177/1754073912445818

[B62] Nie D. Wang X. W. Shi L. C. Lu B. L. (2011). “EEG-based emotion recognition during watching movies,” in Proceedings of the 5th International IEEE/EMBS Conference on Neural Engineering (Cancun, Mexico), 667–670.

[B63] Niedenthal P. M. Winkielman P. Mondillon L. Vermeulen N. (2009). Embodiment of emotion concepts. J. Pers. Soc. Psychol. 96, 1120–1136. 10.1037/a0015574 19469591

[B64] Novak D. Goršič M. Podobnik J. Munih M. (2014). Toward real-time automated detection of turns during gait using wearable inertial measurement units. Sensors 14, 18800–18822. 10.3390/s141018800 25310470 PMC4239865

[B65] Ortony A. Turner T. J. (1990). What’s basic about basic emotions? Psychol. Rev. 97, 315–331. 10.1037/0033-295X.97.3.315 1669960

[B66] Osgood C. E. (1966). Dimensionality of the semantic space for communication via facial expressions. Scand. J. Psychol. 7, 1–30. 10.1111/j.1467-9450.1966.tb01334.x 5908205

[B67] Osinsky R. Karl C. Hewig J. (2017). Dispositional anxiety and frontal-midline theta: on the modulatory influence of sex and situational threat. J. Pers. 85, 300–312. 10.1111/jopy.12241 26773206

[B68] Panksepp J. (2010). Affective consciousness in animals: perspectives on dimensional and primary process emotion approaches. Proc. Biol. Sci. 277, 2905–2907. 10.1098/rspb.2010.1017 20685709 PMC2982033

[B69] Park M. S. Oh H. S. Jeong H. Sohn J. H. (2013). “EEG-based emotion recogntion during emotionally evocative films,” in Proceedings of the 2013 International Winter Workshop on Brain-Computer Interface, Kor, East Azerbaijan, 56–57.

[B70] Poole B. D. Gable P. A. (2014). Affective motivational direction drives asymmetric frontal hemisphere activation. Exp. Brain Res. 232, 2121–2130. 10.1007/s00221-014-3902-4 24658634

[B71] Reuderink B. Hl C. Poel M. (2013). Valence, arousal and dominance in the EEG during game play. Int. J. Auton. Adapt. Commun. Syst. 6, 45–62. 10.1504/ijaacs.2013.050691

[B72] Rohlfs P. Ramírez J. M. (2006). Aggression and brain asymmetries: a theoretical review. Aggress. Violent Behav. 11, 283–297. 10.1016/j.avb.2005.09.001

[B73] Russell J. A. Mehrabian A. (1977). Evidence for a three-factor theory of emotions. J. Res. Pers. 11, 273–294. 10.1016/0092-6566(77)90037-x

[B74] Sammler D. Grigutsch M. Fritz T. Koelsch S. (2007). Music and emotion: electrophysiological correlates of the processing of pleasant and unpleasant music. Psychophysiology 44, 293–304. 10.1111/j.1469-8986.2007.00497.x 17343712

[B75] Sandler H. Tamm S. Fendel U. Rose M. Klapp B. F. Bösel R. (2016). Positive emotional experience: induced by vibroacoustic stimulation using a body monochord in patients with psychosomatic disorders: is associated with an increase in EEG-theta and a decrease in EEG-alpha power. Brain Topogr. 29, 524–538. 10.1007/s10548-016-0480-8 26936595

[B76] Sanei S. Chambers J. A. (2007). EEG signal processing. Comput. Intell. Neurosci. 2007, 1178–1181. 10.1002/9780470511923 PMC2246088 18301719

[B77] Santibanez G. Bloch S. (1986). A qualitative analysis of emotional effector patterns and their feedback. Pavlov J. Biol. Sci. 21, 108–116. 3748633 10.1007/BF02699344

[B78] Schaefer A. Nils F. Sanchez X. Philippot P. (2010). Assessing the effectiveness of a large database of emotion-eliciting films: a new tool for emotion researchers. Cogn. Emot. 24, 1153–1172. 10.1080/02699930903274322

[B79] Schmidt L. A. Trainor L. J. (2001). Frontal brain electrical activity (EEG) distinguishes valence and intensity of musical emotions. Cogn. Emot. 15, 487–500. 10.1080/02699930126048

[B80] Schweiger D. Stemmler G. Burgdorf C. Wacker J. (2014). Opioid receptor blockade and warmth-liking: effects on interpersonal trust and frontal asymmetry. Soc. Cogn. Affect. Neurosci. 9, 1608–1615. 10.1093/scan/nst152 24078107 PMC4187277

[B82] Sjöstrand K. Clemmensen L. H. Larsen R. Ersbøll B. Einarsson G. (2018). SpaSM: a MATLAB toolbox for sparse statistical modeling. J. Stat. Softw. 84:10 10.18637/jss.v084.i10

[B83] Stikic M. Johnson R. R. Tan V. Berka C. (2014). EEG-based classification of positive and negative affective states. Brain Comput. Interfaces 1, 99–112. 10.1080/2326263x.2014.912883

[B84] Suetsugi M. Mizuki Y. Ushijima I. Kobayashi T. Tsuchiya K. Aoki T.. (2000). Appearance of frontal midline theta activity in patients with generalized anxiety disorder. Neuropsychobiology 41, 108–112. 10.1159/000026641 10644932

[B85] Takahashi H. Matsuura M. Koeda M. Yahata N. Suhara T. Kato M.. (2008). Brain activations during judgments of positive self-conscious emotion and positive basic emotion: pride and joy. Cereb. Cortex 18, 898–903. 10.1093/cercor/bhm120 17638925

[B86] Thibodeau R. Jorgensen R. S. Kim S. (2006). Depression, anxiety and resting frontal EEG asymmetry: a meta-analytic review. J. Abnorm. Psychol. 115, 715–729. 10.1037/0021-843x.115.4.715 17100529

[B87] Vecchiato G. Toppi J. Astolfi L. Fallani F. D. Cincotti F. Mattia D.. (2011). Spectral EEG frontal asymmetries correlate with the experienced pleasantness of TV commercial advertisements. Med. Biol. Eng. Comput. 49, 579–583. 10.1007/s11517-011-0747-x 21327841

[B88] Verma G. K. Tiwary U. S. (2014). Multimodal fusion framework: a multiresolution approach for emotion classification and recognition from physiological signals. Neuroimage 102, 162–172. 10.1016/j.neuroimage.2013.11.007 24269801

[B89] Wacker J. Heldmann M. Stemmler G. (2003). Separating emotion and motivational direction in fear and anger: effects on frontal asymmetry. Emotion 3, 167–193. 10.1037/1528-3542.3.2.167 12899417

[B90] Wacker J. Mueller E. M. Pizzagalli D. A. Hennig J. Stemmler G. (2013). Dopamine-d2-receptor blockade reverses the association between trait approach motivation and frontal asymmetry in an approach-motivation context. Psychol. Sci. 24, 489–497. 10.1177/0956797612458935 23447558

[B91] Wilkowski B. M. Meier B. P. (2010). Bring it on: angry facial expressions potentiate approach-motivated motor behavior. J. Pers. Soc. Psychol. 98, 201–210. 10.1037/a0017992 20085395

[B92] Yani-de-Soriano M. M. Foxall G. R. (2006). The emotional power of place: the fall and rise of dominance in retail research. J. Retail. Consum. Serv. 13, 403–416. 10.1016/j.jretconser.2006.02.007

[B93] Yin D. Bond S. D. Zhang H. (2014). Anxious or angry? Effects of discrete emotions on the perceived helpfulness of online reviews. MIS Q. 38, 539–560. 10.25300/misq/2014/38.2.10

[B94] Yoon H. J. Chung S. Y. (2011). “EEG spectral analysis in valence and arousal dimensions of emotion,” in Paper presented at the International Conference on Control, Automation and Systems (Gyeonggi-do, South Korea), 1319–1322.

[B95] Zhao G. Ge Y. Shen B. Wei X. Wang H. (2017). Emotion analysis for personality inference from EEG signals. IEEE Trans. Aff. Comp. 9, 362–371. 10.1109/taffc.2017.2786207

